# Application of shotgun metagenomics sequencing and targeted sequence capture to detect circulating porcine viruses in the Dutch–German border region

**DOI:** 10.1111/tbed.14249

**Published:** 2021-08-28

**Authors:** Leonard Schuele, Erley Lizarazo‐Forero, Katrin Strutzberg‐Minder, Sabine Schütze, Sandra Löbert, Claudia Lambrecht, Jürgen Harlizius, Alex W. Friedrich, Silke Peter, John W. A. Rossen, Natacha Couto

**Affiliations:** ^1^ Department of Medical Microbiology and Infection Prevention University Medical Center Groningen, University of Groningen Groningen The Netherlands; ^2^ Institute of Medical Microbiology and Hygiene University of Tübingen Tübingen Germany; ^3^ IVD Innovative Veterinary Diagnostics (IVD GmbH) Seelze Germany; ^4^ Animal Health Services Chamber of Agriculture of North Rhine‐Westphalia Bad Sassendorf Germany; ^5^ Department of Pathology University of Utah School of Medicine Salt Lake City Utah USA; ^6^ The Milner Centre for Evolution, Department of Biology and Biochemistry University of Bath Bath UK

**Keywords:** influenza A virus, one health, porcine virome, shotgun metagenomics sequencing, surveillance, targeted sequence capture

## Abstract

Porcine viruses have been emerging in recent decades, threatening animal and human health, as well as economic stability for pig farmers worldwide. Next‐generation sequencing (NGS) can detect and characterize known and unknown viruses but has limited sensitivity when an unbiased approach, such as shotgun metagenomics sequencing, is used. To increase the sensitivity of NGS for the detection of viruses, we applied and evaluated a broad viral targeted sequence capture (TSC) panel and compared it to an unbiased shotgun metagenomic approach. A cohort of 36 pooled porcine nasal swab and blood serum samples collected from both sides of the Dutch–German border region were evaluated. Overall, we detected 46 different viral species using TSC, compared to 40 viral species with a shotgun metagenomics approach. Furthermore, we performed phylogenetic analysis on recovered influenza A virus (FLUAV) genomes from Germany and revealed a close similarity to a zoonotic influenza strain previously detected in the Netherlands. Although TSC introduced coverage bias within the detected viruses, it improved sensitivity, genome sequence depth and contig length. In‐depth characterization of the swine virome, coupled with developing new enrichment techniques, can play a crucial role in the surveillance of circulating porcine viruses and emerging zoonotic pathogens.

## 1 INTRODUCTION

The emergence of new viral diseases poses a continuous threat to both animal and human health. Wildlife‐borne diseases such as Lassa fever (Roberts, 2018) and West Nile fever (Vlaskamp et al., 2020) and those linked to livestock such as avian and swine influenza (Fraaij et al., 2016; Lam et al., 2015) have emerged previously and have caused significant epidemics/pandemics with serious repercussions. With the increasing intensification of livestock farming, a rise in not only the human–wildlife–livestock interface, but also within herds, has led to an increased risk of transmission (Jones et al., 2013; Kwok et al., 2020). Therefore, the surveillance of farms and the environment is critical for detecting (emerging) zoonotic infectious diseases.

Pigs are the most commonly studied farm animals as they are considered mixing vessels in the transmission of epidemic/pandemic viruses (Smith et al., 2009). The 2009 swine‐origin H1N1 influenza A virus (FLUAV), which was derived from co‐circulating FLUAV strains in swine, was initially transmitted to humans several months before the outbreak was identified (Smith et al., 2009). The results of several studies highlight the need for systematic surveillance of FLUAV in swine. Additionally, these studies can provide evidence of reassortment of co‐circulating viruses in swine, leading to the emergence of potentially pandemic viruses in humans (Nava et al., 2009). Moreover, pigs can also be affected by several swine‐specific viruses, for example the African swine fever virus (Taylor et al., 2020) and porcine reproductive and respiratory syndrome virus (PRRSV) (Balka et al., 2018), that can cause severe production losses. Lastly, characterization and understanding of the pig virome are also essential when assessing the safety of xenotransplant development (Denner, 2017).

Next‐generation sequencing (NGS) has been used previously to identify and characterize viruses (Lizarazo et al., 2019). Shotgun metagenomics sequencing (SMg) depicts the untargeted sequencing of nucleic acids directly from the sample. SMg has the potential for broad range detection, characterization and detailed taxonomic classification of pathogens, making it a promising tool within a One Health approach (Wylie et al., 2015). As such, SMg has been used to detect and characterize known and novel viruses affecting plants, humans and animals (Kwok et al., 2020; Palinski et al., 2017). Furthermore, SMg can detect co‐infections and provide genomic data for epidemiological typing (Couto et al., 2018). However, the inherent unspecific nature of SMg results in the sequencing of host, environmental, pathogenic and non‐pathogenic nucleic acids, which results in an overall lower sensitivity, compared to conventional methods such as real‐time PCR (Quick et al., 2017). Therefore, sensitivity is not only determined by the abundance of microorganisms but more so by the presence of host cells and other microbes (Couto et al., 2018). To improve the sensitivity of microbe detection, several pre‐ and post‐lysis enrichment strategies have been described. Pre‐lysis enrichment depends on the microorganisms’ structural integrity, as it involves targeted lysis of host cells followed by degradation of free nucleic acids (Hasan et al., 2016) and/or separation by centrifugation/filtration (Bellehumeur et al., 2015). Post‐lysis enrichment steps include DNase treatment (Lizarazo et al., 2019), oligonucleotide bait probes (targeted sequence capture [TSC]) (Oba et al., 2018; Wylie et al., 2015), rRNA depletion and PCR amplicon sequencing (Quick et al., 2017). Oligonucleotide bait probes capture viral nucleic acids present in a sample by hybridization and have been reported to be superior to other pre‐lysis and post‐lysis enrichment methods at increasing the number of sequenced viral reads, while maintaining viral diversity (Briese et al., 2015). As a result, viral TSC was selected to be evaluated in the sequencing of highly diverse pig samples.

One goal of the Food Protects project (https://www.foodprotects.eu/projekt/arbeitspakete/tic2/) was to improve early warning of infectious diseases through innovative technology. In the current study, we compared two NGS‐based approaches: an unbiased shotgun metagenomics based on Sequence‐Independent Single‐Primer‐Amplification (SISPA) technique and a targeted viral capture panel (ViroCap) (Wylie et al., 2015) on 36 pooled pig samples. Samples from a cohort consisting of blood serum (BS) and nasal swabs (NS) covering various viral loads (previously determined by qPCR) were selected for the comparison of the two protocols. In this study, we aimed to (i) optimize the ViroCap protocol, (ii) assess ViroCap compared to shotgun metagenomics sequencing and (iii) detect circulating porcine viruses in a significant pig farming cluster in Europe. Different bioinformatics tools were applied. This study demonstrates the potential of NGS approaches to understand the phylogeny of important human and animal viruses circulating in pig farms.

## 2 MATERIALS AND METHODS

### 2.1 Sample collection, qPCR and nucleic acid isolation

**FIGURE 1:**
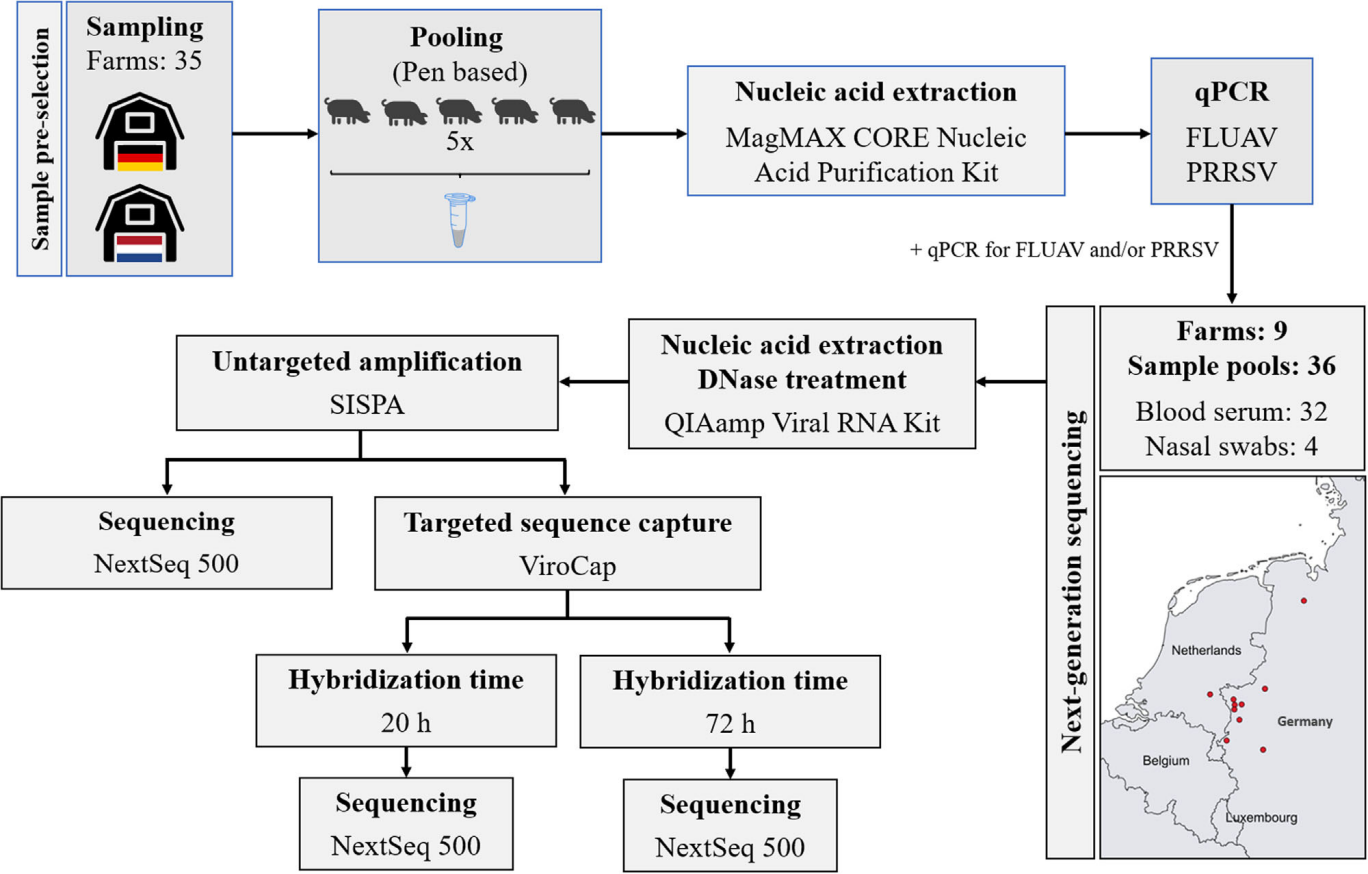
Flow chart of the study design. Samples were first pre‐selected based on positive qPCR results for FLUAV and/or PRRSV. Subsequent NGS analysis using both a metagenomics and a targeted sequence capture approach was performed. For viral targeted sequence capture using ViroCap, two different hybridization times were evaluated. The relative location of the selected farms for NGS analysis is indicated as red dots on the map.

Between October 2017 and 2018, BS and NS samples were collected from 35 farms in the Dutch–German border region under the Food Protects project (Figure 1). To monitor for circulating viruses on the herd level, pools were created by combining samples from five animals within the same herd and age group. qPCR specific for PRRSV (Virotype^®^ PRRSV RT‐PCR Kit, Indical Bioscience, Leipzig, Germany) and FLUAV (VetMAX™‐Gold SIV Detection Kit, Life Technologies, Carlsbad, CA, USA) were performed on the pooled samples following the manufacturers’ recommendations. A total of 36 sample pools (with varying Ct‐values) from nine farms (Figure 1) were selected for NGS analysis based on a positive FLUAV and/or PRRSV qPCR result: 32 BS pools and four NS pools. The available metadata for each farm is present in Table 1

**TABLE 1:** Overview of farms selected for sequencing

Farm ID	Pooled sample type	Ct PRRSV	Ct SIV^a^	Clinical symptoms	Sampling date	Number of pigs per farm	Age	Sample ID
1 (*n* = 5)	BS	26	cNS: Neg	None	September 2017	3300	Pre‐fattening	251 (1–5)
2 (*n* = 2)	BS	23	cNS: Neg	None	September 2017	1900	Pre‐fattening	254 (3,5)
3 (*n* = 10)	BS	25 28	cNS: Neg	Respiratory	October 2017	230	Pre‐fattening	278 (1, 3–5) 278 (6–10)
4.1 (*n* = 4)	BS	28	cNS: 34	NA	October 2017	NA	Pre‐fattening	313 (7–10)
4.2 (*n* = 3)	BS	28	cNS: Neg				Mid‐fattening	313 (11–13)
5.1 (*n* = 1)	NS	NA	19	Respiratory, enteral (closed system)	October 2018	NA	Pre‐fattening	213–14
5.2 (*n* = 1)	NS	NA	20					213–15
5.3 (*n* = 1)	NS	NA	26				Mid‐fattening	213–16
5.4 (*n* = 1)	NS	NA	22					213–17
6 (*n* = 2)	BS	23	cNS: Neg	Respiratory	September 2018	NA	Piglets (20 kg)	213 (19, 20)
7 (*n* = 3)	BS	24	cNS: Neg	NA	October 2018	NA	Piglets (9–13 weeks)	213 (21–23)
8 (*n* = 2)	BS	26	cNS: Neg	NA	October 2018	NA	Piglets (15–20 kg)	213 (24, 25)
9 (*n* = 2)	BS	29	cNS: Neg	Respiratory	September 2018	2400	Pre‐fattening	213 (26, 27)

^a^ In case the pooled sample type is BS, the Ct values for SIV refer to the corresponding nasal swabs (cNS) collected from the same animals.

Abbreviations: BS, blood serum; cNS, corresponding nasal swab; Ct, cycle threshold; NA, not available; Neg, negative; NS, nasal swab; PRRSV, porcine reproductive and respiratory syndrome virus; SIV, swine influenza virus.

### 2.2 Next‐generation sequencing

Total nucleic acids were extracted from 140 μl of sample material with the QIAamp Viral RNA Mini Kit (Qiagen) and eluted in 90 μl. The eluate was then subjected to TurboDNase (ThermoFisher Scientific, Waltham, USA) treatment according to the manufacturer's instructions and concentrated with the RNA Clean & Concentrator‐5 kit (Zymo Research, Irvine, USA). Complementary DNA (cDNA) was generated using a SISPA approach as described previously (Kafetzopoulou et al., 2018). Briefly, reverse transcription and synthesis of second‐strand cDNA were performed as described (Greninger et al., 2015). Amplification of cDNA was performed as described (Kafetzopoulou et al., 2018) using Sol‐Primer B (5′‐GTTTCCCACTGGAGGATA‐3′) and the following PCR reaction conditions: 98°C for 30 s; 30 cycles of 94°C for 15 s, 50°C for 20 s and 68°C for 3 min, followed by 68°C for 10 min. The amplified cDNA was cleaned with a 1:0.5 ratio of AMPure XP beads (Beckman Coulter, Brea, CA, USA). Sequencing libraries were generated with the KAPA HyperPlus Kit (Roche, Basel, Switzerland) according to the manufacturer's recommendations. The SISPA‐generated libraries were used as a basis for the unbiased SMg approach (hereafter named SISPA approach) and for the viral targeted capture panel (hereafter named ViroCap approach). Viral capture was performed with the ViroCap share developer panel (Roche), according to SeqCap EZ HyperCap Workflow User's Guide v2.1. ViroCap consists of approximately 2 million capture probes derived from vertebrate viral genomes known in 2014 (Wylie et al., 2015). The oligonucleotide capture probes hybridize with target viral nucleic acids and separate from the background with magnetic streptavidin‐coated beads. Two hybridization times were initially tested, 20 and 72 h. For both approaches, 12 samples and a negative control consisting of lysis buffer were sequenced on an Illumina NextSeq 500 (2 × 76 bp) using the v2.5 mid‐output chemistry (Illumina, San Diego, CA, USA). Recommended combinations of KAPA dual‐indexed adapters (Roche) were selected to reduce crosstalk.

### 2.3 Data analysis

Adapter and quality trimming (error probability threshold of 0.01, corresponding to a Phred score threshold of 20) was performed in CLC Genomics Workbench v12.0.3 (CLC) (Qiagen, Aarhus, Denmark). To obtain read‐based taxonomic identification and binning, trimmed reads were uploaded onto Taxonomer (Flygare et al., 2016) and run on full analysis mode. A relative read count threshold of 0.01% was applied to eliminate low target viral reads and account for possible barcode contamination (O'Flaherty et al., 2018). Read normalization was generated from CLC. Reads were mapped against the *Sus scrofa* reference genome v11.1 to remove host sequences. Unmapped reads were assembled with CLC, SPAdes v3.13.1 (metagenomics mode) (Bankevich et al., 2012) and MEGAHIT v1.2.8 (Li et al., 2015), keeping only contigs ≥200 bp. Assembly metrics were compared using QUAST v5 (Gurevich et al., 2013). MEGAHIT assemblies were mapped (80% identity, 80% length fraction, ignore unspecific reads) against an in‐house viral database derived from available complete genomes on GenBank on 13 August 2019 using CLC. Consensus sequences were also manually aligned using BLASTn on NCBI and contigs had to map to at least two regions of the respective reference genome to be considered valid. A Student's *t*‐test was performed (*p* < 0.05) to determine if there were any significant differences in the proportion of viral sequence reads between ViroCap hybridization times.

Phylogenetic reconstruction was performed with the MEGAHIT assemblies that achieved nearly full‐length genomes. PRRSV genomes from this study (*n* = 16), together with relevant, complete genome sequences retrieved from the NCBI database (*n* = 26), were used. The sequences were aligned with MAFFT (Katoh et al., 2002). Regression of sampling time versus root‐to‐tip genetic distance was performed using TempEst v1.5.1 to investigate the alignments’ temporal signal and data quality (Rambaut et al., 2016). To create a FLUAV phylogenetic tree, 7620 genomes between 2015 and 2020 (1878 swine host and 5742 human host) were retrieved from the Influenza Research Database (https://www.viprbrc.org/). Representative haemagglutinin sequences from different clusters were obtained through CD‐hit software with a cutoff of ≤97% identity and aligned with MAFFT (Katoh et al., 2002). The phylogenetic trees were then inferred from the alignments using the maximum likelihood approach implemented in RA×ML v8.2.10 (Stamatakis, 2014) under the General Time Reversible (GTR) CAT substitution model (Stamatakis, 2014) and rapid bootstrapping from 1000 replicates. The phylogenetic analysis was carried out on the freely available CIPRES Science Gateway v3.3 portal www.phylo.org (Miller et al., 2012). The in silico Influenza Antiviral Resistance Risk Assessment was performed on www.fludb.org.

## 3 RESULTS

### 3.1 Impact of probe hybridization time on viral sensitivity

**FIGURE 2:**
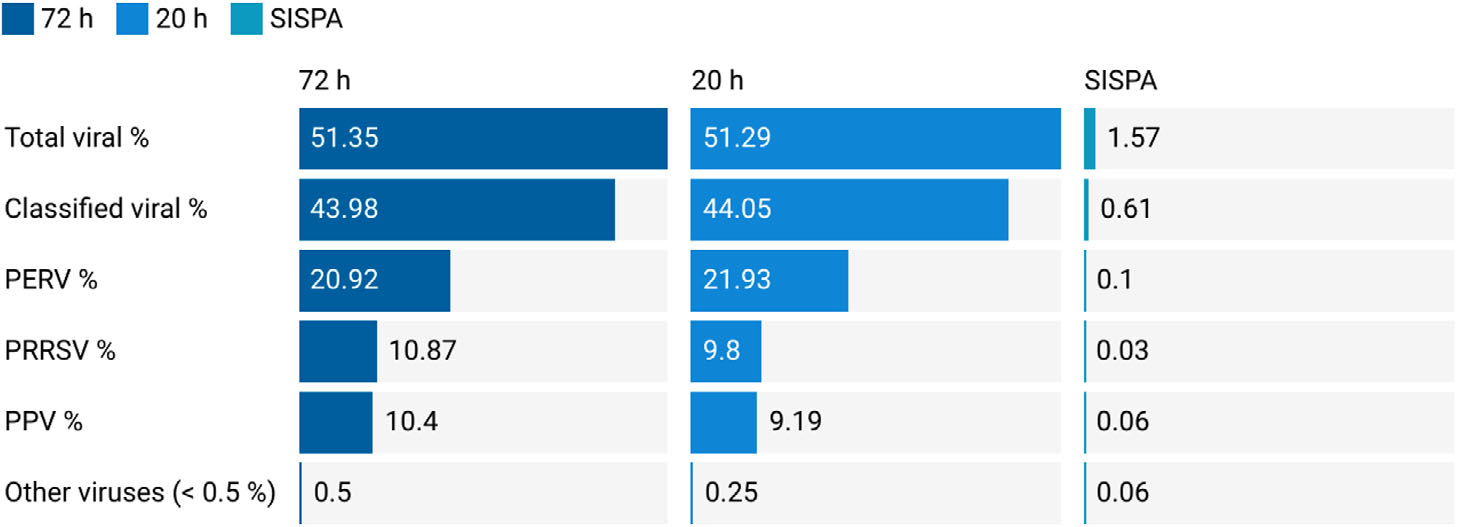
Impact of ViroCap hybridization times (20 and 72 h) on viral sensitivity compared to SISPA (*n* = 12 samples). The diagram highlights the most frequently detected viruses. Sequencing reads were analyzed with Taxonomer (full analysis) and normalized. Abbreviations: PERV, porcine endogenous retrovirus; PRRSV, porcine reproductive and respiratory syndrome virus; PPV, porcine parvovirus.

To set up an efficient viral enrichment strategy for ViroCap, hybridization times of 20 and 72 h were compared on a subset of 12 BS samples (Figure 2). SISPA served as a baseline.

A hybridization time of 20 h resulted in an overall viral read count of 51.29%, whereas 72 h resulted in a slight increase to 51.35%. Both 20 h and 72 h resulted in a 32.7‐fold change increase of viral reads compared to SISPA (1.57% viral reads). The percentages of classified viral reads obtained with different hybridization times were similar and resulted in a 72.34‐ and 72.46‐fold change in viral reads, compared to SISPA (classified viral reads 0.61%) for 20 and 72 h hybridization times, respectively. As there was no significant difference (*p*‐value = 0.996) between the two hybridization times, a hybridization time of 20 h was selected to proceed.

### 3.2 Comparison of viral sensitivity between SISPA and ViroCap

**FIGURE 3:**
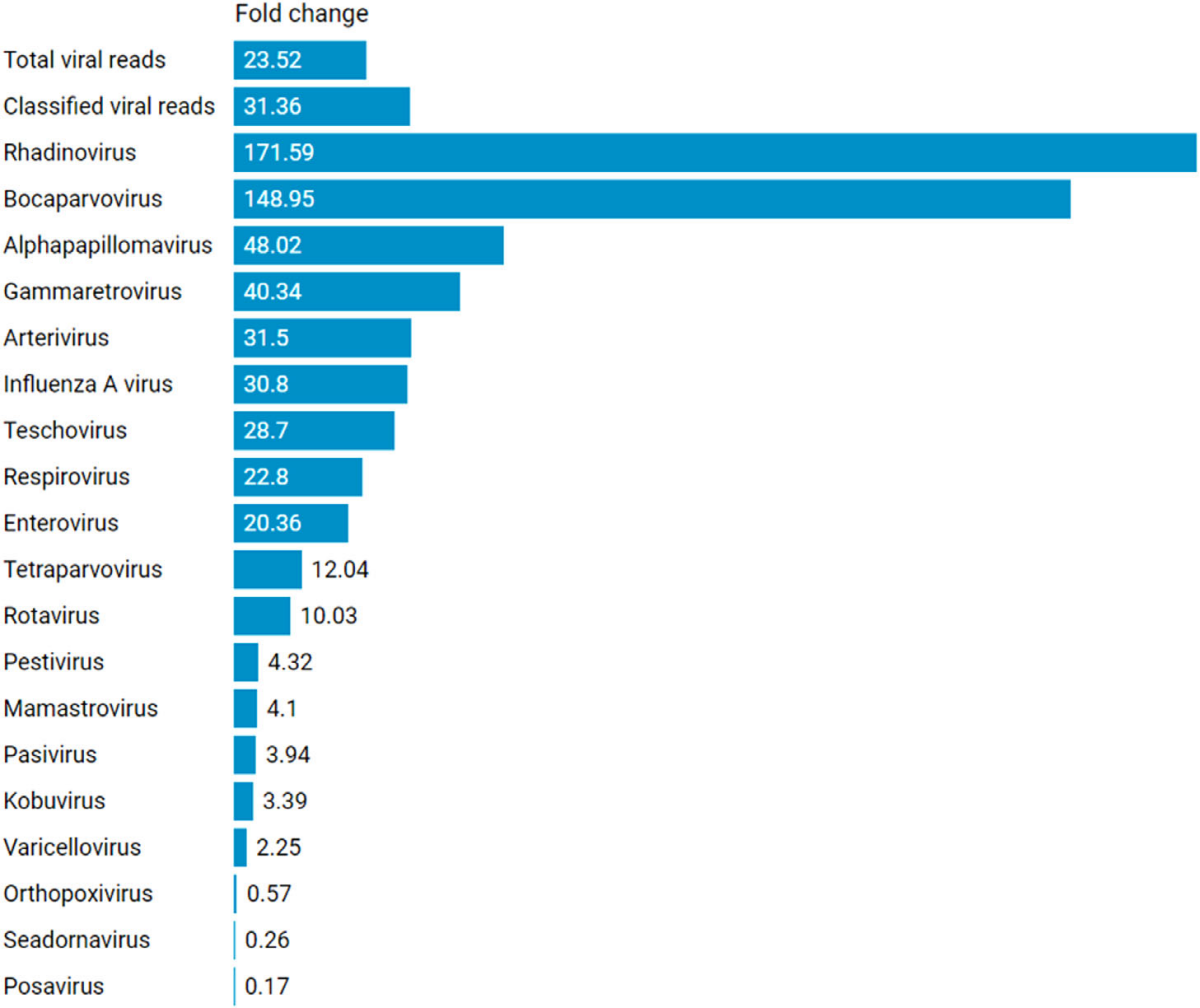
Viral reads (normalized) and fold changes between SISPA and ViroCap (*n* = 36 samples). Frequently detected viral genera in this study are shown. Numbers higher than 1 indicate increased sensitivity using ViroCap. Data analyzed with Taxonomer (full analysis).

In total, 36 samples (32 BS and four NS) were evaluated using SISPA and ViroCap to compare viral sensitivity. Using the kmer‐based online tool Taxonomer, a total of 87 viral species were detected with SISPA, and a total of 93 viral species were detected using ViroCap. Viruses detected within each herd and farm using read‐based taxonomic classification are listed in Table S1. Additionally, ViroCap increased the overall viral read count by a fold of 23.5, compared to the SISPA approach alone (Figure 3; Table S2). The relationship between FLUAV Ct values and the number of FLUAV reads is shown in Table S3. No significant association or correlation was found between these two parameters.

Overall, ViroCap led to a higher read count in 16 of the 19 most abundantly detected viral genera in this study. The most significant fold change occurred in rhadinoviruses (171.59‐fold change). There was a loss of viral read count in three viral genera, as shown in Figure 3.

### 3.3 De novo assembly of SISPA and ViroCap sequencing reads

**FIGURE 4:**
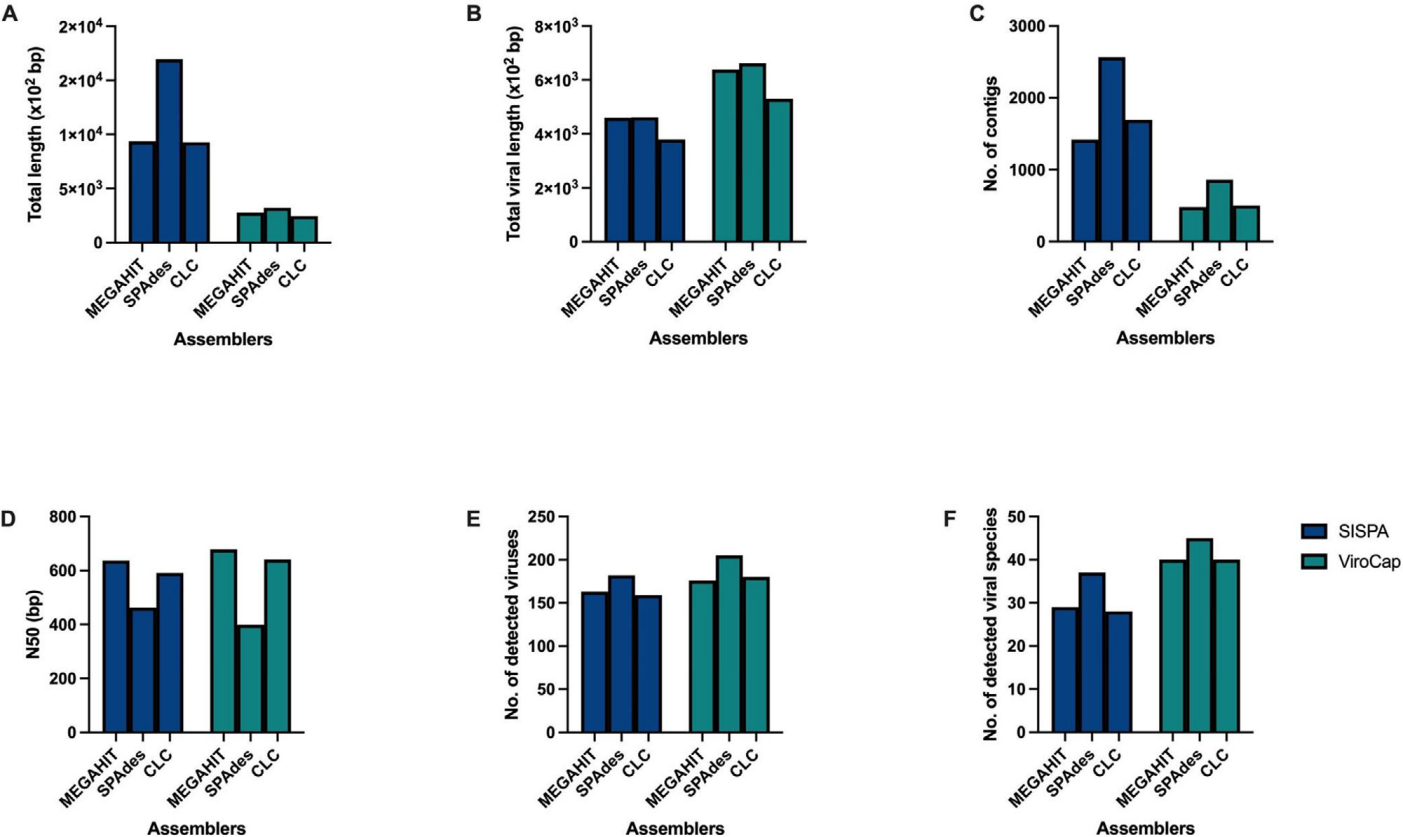
Comparison of MEGAHIT, SPAdes and CLC assemblies (using SISPA and ViroCap). Assembly metrics: (a) Total length (sum of all contigs in bp); (b) Total viral length (sum of all viral contigs in bp); (c) Total number of contigs; (d) N50 (bp); (e) Total number of detected viruses; (f) Total number of detected viral species.

Three de novo assembly tools, MEGAHIT, SPAdes and CLC, were used to analyze the SISPA and ViroCap sequence data and their assembly metrics were compared (Figure 4).

Overall, MEGAHIT yielded the longest contig length and the highest N50 value with the lowest number of contigs, both in SISPA and ViroCap. SPAdes yielded the longest combined assembly (total length) and identified the highest number of viruses in SISPA and ViroCap: 37 with SISPA and 45 with ViroCap. CLC assemblies led to the detection of 28 different viral species with SISPA and 40 viral species with ViroCap. Similarly, MEGAHIT assemblies led to the detection of 29 viral species with SISPA and 40 viral species with ViroCap. Overall, a total of 40 viral species were detected with SISPA and 46 viral species with ViroCap (Figure 4b).

### 3.4 Contig‐based detection of clinically relevant pathogens

In Table 2, we show 37 different viruses with relevance to vertebrates. Overall, 18 viruses were detected in the four NS and 29 viruses in the 32 BS samples. *Astroviridae*, *Arteriviridae*, and *Flaviviridae* were the most frequently detected viral families. Multiple pig pathogenic viruses were detected, such as porcine astroviruses, porcine bocaviruses, PRRSV and porcine pestiviruses. Furthermore, we detected viruses with zoonotic potential, such as FLUAV (four samples), norovirus GII.2/pig (one sample) and porcine rotavirus A/C (three samples). In sample 213–24, we were able to partially recover four segments of a porcine rotavirus A (Table S4; Figure S1). Viruses detected in pigs suffering from respiratory symptoms are listed in Table S5 (NS) and Table S6 (BS). Meanwhile, viruses detected in animals without symptoms are listed in Table S7 (BS). Additional complete or near‐complete viral contigs obtained in this study are shown in Table S8.

**TABLE 2:** Overview of detected viruses (contig level) in the respective sample material and associated symptoms/pathology: Red (blood serum), green (nasal swab) and blue (blood serum and nasal swab)

	(*n* = 36)		
Virus	SISPA	ViroCap	Pig‐associated symptoms/pathology (and remarks)
Astrovirus wild boar WBAstV‐1	8	11	Associated with gastroenteritis. Can be found in boars worldwide (Vilcek et al., 2019).
Atypical porcine pestivirus 1	11	10	Associated with neurological disorders (congenital tremors). Can cause disease in piglets (Gatto et al., 2019).
Bocavirus pig/SX/China/2010	2	2	Trembling, fever, testicular atrophy, abortion or death. Symptoms are often associated with co‐infections (Zhou et al., 2014).
Hubei tombus‐like virus 8	7	3	Unknown host and pathology, considered plant virus, but host spectrum could be broader (hedgehogs) (Reuter et al., 2019).
Influenza A (H1N1) virus	4	4	Fever and respiratory symptoms. Zoonotic potential (Fraaij et al., 2016).
PRRSV (Lelystad)	24	26	Reproductive failure, abortions, respiratory distress. Tremendous economic burden for pig farms (Bellehumeur et al., 2015).
Mamastrovirus 2	4	8	Nervous system disease. Found in humans, pigs, cattle and mink (Chen et al., 2020).
Mamastrovirus 3	0	1	
Norovirus pig/DO35/KOR	0	2	Acute gastroenteritis in humans and animals; zoonotic transmission possible (Wang et al., 2005).
Parvovirus YX‐2010/CHN	1	4	As yet, non‐pathogenic virus (Wang et al., 2010).
Pasivirus A1	1	2	Unknown pathology. Pigs serve as natural hosts (Hanke et al., 2017).
Porcine astrovirus 2	1	3	Gastrointestinal disease, neurological disease. High genetic diversity and variability. Unclear zoonotic potential. Report of porcine–human recombinants with transmission from humans to pigs (De Benedictis et al., 2011).
Porcine astrovirus 3	1	1	
Porcine astrovirus 4	5	8	
Porcine astrovirus 5	0	1	
Porcine bocavirus 5/JS677	0	1	Trembling, fever, testicular atrophy, abortion or death. Symptoms are often associated with co‐infections (Zhou et al., 2014).
Porcine bocavirus H18	3	3	
Porcine enteric sapovirus	0	1	Gastroenteritis (Proietto et al., 2016).
PERV	32	32	As yet, non‐pathogenic virus. Potential safety risk in porcine xenotransplantations (Denner, 2017).
Porcine enterovirus 9	1	1	Mostly asymptomatic. Occasional pneumonia and enteric disease. Isolated from healthy pigs in Asia and Europe (Anbalagan et al., 2014).
Porcine hokovirus	1	8	Unknown. Spread in pigs and wild boars (Adlhoch et al., 2010).
Porcine kobuvirus	0	5	Suspected cause of diarrhoea in piglets. Continental spread in wild boar populations (Proietto et al., 2017).
Porcine kobuvirus SH‐W‐CHN/2010/China	1	3	
Porcine kobuvirus swine/S‐1‐HUN/Hungary	1	3	
Porcine lymphotropic herpesvirus 2	1	1	Postweaning multisystemic wasting syndrome. Latent virus, high prevalence in pigs (McMahon et al., 2006).
Porcine pestivirus 1	4	4	Congenital tremors, neurological disorders. Found in North/South America, Europe and Asia (Gatto et al., 2019).
Porcine respirovirus 1	1	1	Potential role in respiratory disease. Initially detected in deceased pigs in Hong Kong (Lau et al., 2013).
Porcine sapelovirus 1	3	4	Encephalitis, reproductive disorders, respiratory distress and skin lesions. Closely related to the genus Enterovirus (Piorkowski et al., 2017).
Porcine torovirus	1	2	Potential enteric swine pathogen, high rate in piglets. First detected in the Netherlands (1998), now emerged in many countries (Hu et al., 2019).
Rotavirus A	0	2	Gastroenteritis in humans and animals (Vlasova et al., 2017).
Porcine Rotavirus C	2	3	
Teschovirus A	1	3	Mostly asymptomatic, can lead to teschovirus encephalomyelitis in pigs (Deng et al., 2012)
Torque teno sus virus 1b	1	2	Pathogenic role is controversial, might worsen the progression of other diseases. Can be found worldwide in pigs (Lee et al., 2015)
Torque teno sus virus k2a	0	2	
Ungulate tetraparvovirus 3	1	3	Detection in lung samples suggests a pathological role in disease. Pigs are likely the main reservoir (Cságola et al., 2016)
Posavirus 1	1	0	Unknown clinical relevance, aquatic host is likely (Hause et al., 2016)
Posavirus sp.	2	0	

Abbreviations: PERV, porcine endogenous retrovirus; PRRSV, porcine reproductive and respiratory syndrome virus.

Additionally, a total of 399 bacterial species were detected on the contig level. Notably, we found contigs classified by BLASTn as organisms of clinical interest such as *Mycoplasma hyopneumoniae* (one sample), *Salmonella enterica* (one sample), *Bacillus cereus* (seven samples), *Streptococcus suis* (two samples), *Staphylococcus aureus* (five samples) and *Acinetobacter johnsonii/baumannii* (nine samples). Furthermore, we found genes expressed by bacteria that have been associated with disease in other animals, such as *Moraxella bovoculi* (four samples; cattle; Angelos et al., 2007), *Mycoplasma haemocanis* (six samples; dogs; Lashnits et al., 2019), *Riemerella anatipestifer* (four samples; ducks; Zhu et al., 2018) and *Brucella melitensis* (one sample; sheep and goats; Zhang et al., 2018).

### 3.5 PRRSV genome coverage

**FIGURE 5:**
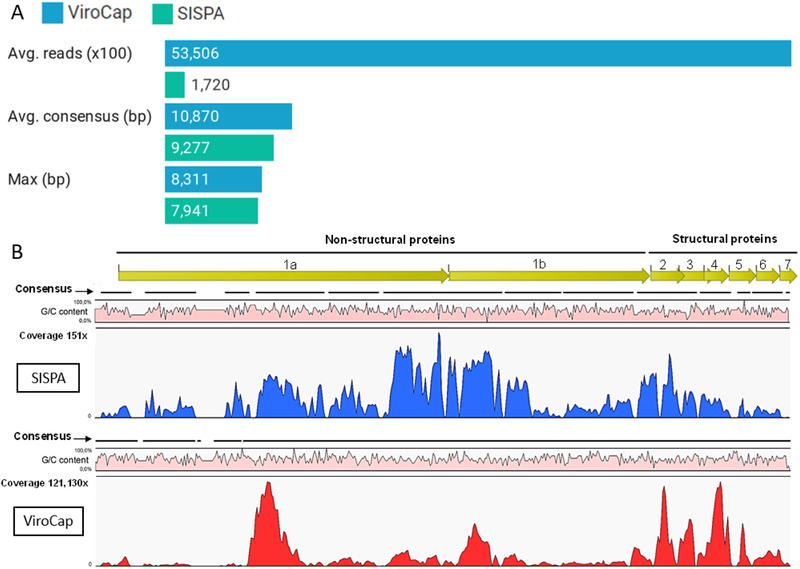
(a) Average PRRSV read count, average contig consensus length and average longest contig. The PRRSV genome size is approximately 15 kb. (b) An example of a genome‐wide comparison of sequence coverage and G/C content of a PRRSV genome using SISPA and ViroCap. The proportion of G/C content (scale 0%–100%) is shown in pink; the sequencing depth coverage is shown in blue for SISPA and red for ViroCap

PRRSV was most frequently detected following assembly, with 26 and 24 samples generating contigs with the ViroCap and SISPA approach, respectively. Coupled with its high abundance and clinical significance, PRRSV was subsequently evaluated in more detail. MEGAHIT assemblies were used as they produced the longest contigs (Figure 4). ViroCap increased the average coverage, along with the number of reads in all 26 samples. Although ViroCap generated more PRRSV reads, the length of the contigs was only slightly longer compared to the PRRSV contigs obtained by SISPA, indicating a coverage bias. Figure 5a illustrates the difference between SISPA and ViroCap in the average number of PRRSV reads (from all samples), the sum of all PRRSV contigs (from all samples) and the (average) longest PRRSV contig (from all samples). Reads from a selected PRRSV contig were then mapped against the closest PRRSV genome from the NCBI database to demonstrate this coverage bias (Figures 5b and S2).

### 3.6 Phylogenetic analysis of PRRSV and FLUAV

ViroCap increased the sequencing depth of two clinically and economically significant viruses, PRRSV and FLUAV. In the following two case studies, we used high‐quality MEGAHIT assemblies generated through ViroCap for epidemiologic analysis.

**FIGURE 6:**
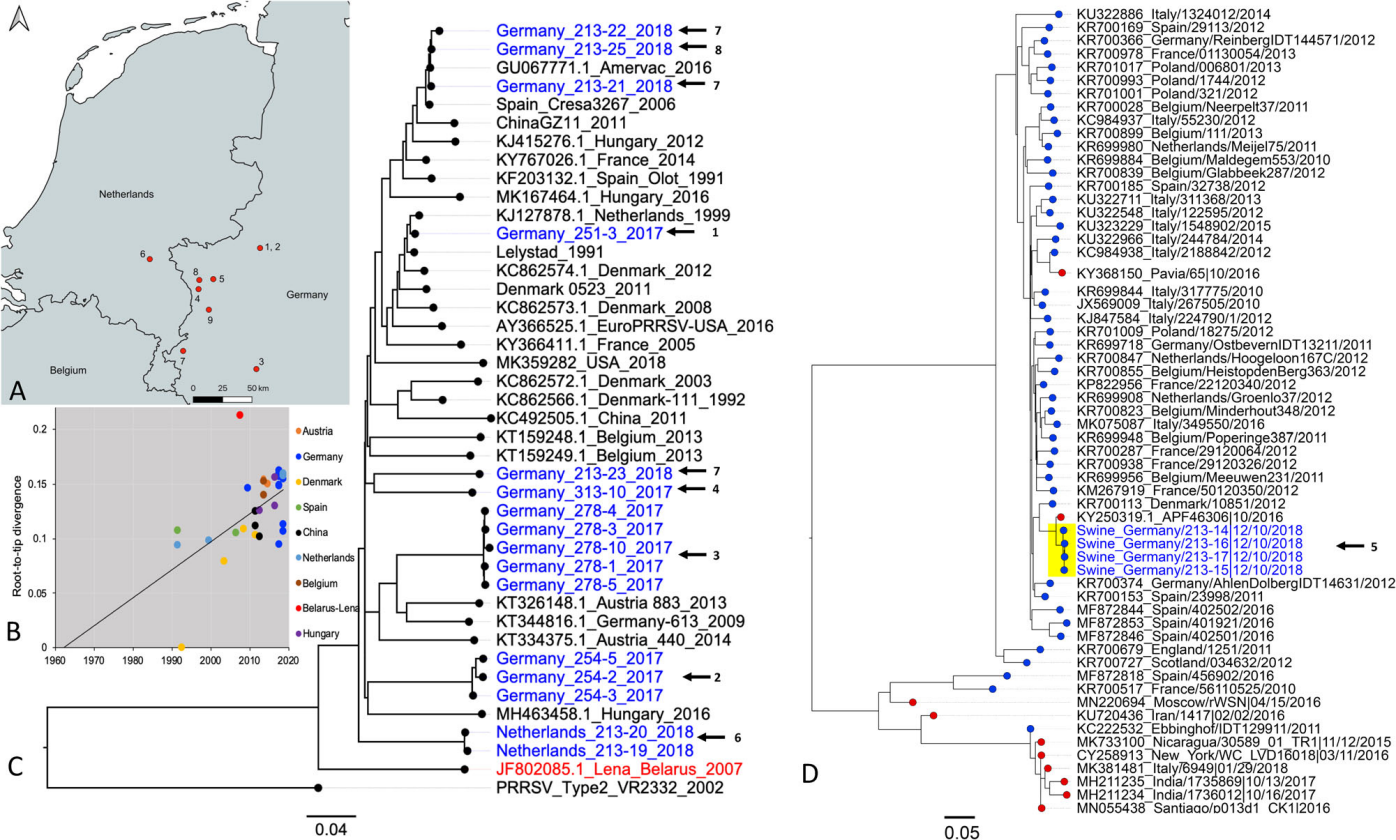
Phylogenetic reconstruction of PRRSV and FLUAV. (a) Map indicating the geographical origin of the samples in this study. Please note, for privacy reasons, the numbers do not correlate with the farm ID in Table 1. (b) Regression of sequence sampling dates against root‐to‐tip genetic distances from the maximum likelihood tree. (c) Phylogenetic analysis of the whole genome of PRRSV. PRRSV 2 prototype strain VR2332 (AY150564) was used as an outgroup. Blue coloured taxa depict the samples from this study. (d) Phylogenetic analysis of the HA sequence from FLUAV. The analysis involved influenza A viruses from swine‐origin from 2010 to 2020 (blue dots) and influenza A viruses isolated from humans from 2015 to 2020 (red dots). A total of 61 representative sequences were used to generate the phylogenetic reconstruction. Samples in this study are highlighted in yellow. The evolutionary history was inferred using the maximum likelihood method implemented in RA×ML with bootstrapping of 1000 replicates

Maximum likelihood trees based on the obtained genomes (∼15 kb) were inferred to estimate PRRSV's evolutionary history. The estimated whole‐genome evolutionary rate was 2.57 × 10^−3^, consistent with other estimates for this virus type (Balka et al., 2018). The tree topology shows that our 16 genomes clustered into three different groups (Figure 6a). Nevertheless, all samples belonged to lineage 1 of PRRSV1. Our results also revealed several samples clustered together with the attenuated virus used in the AMERVAC vaccine (samples 213–21, 213–22, 213–25). Moreover, in two geographic locations (Figure 6a; red dots 7 and 8), we detected both a vaccine‐related strain and a wild‐type strain. Additionally, none of the genomes recovered were closely related to virulent strains (i.e. KJ415276, JF802085). A root‐to‐tip regression estimated the time to the most recent common ancestor to be 1962 (Figure 6b). The root‐to‐tip divergence regression also showed the vaccine‐related strains’ effect (low‐rate variation under the regression line) on the whole viral population's evolutionary rate. Lastly, we found wild‐type strains with higher variation rate in vaccinated pigs.

FLUAV was detected in all four NS samples. The samples originated from pigs of the same farm that had respiratory symptoms (Figure 6d). An NCBI BLASTn search of all viral genome segments revealed a FLUAV strain previously isolated in the Netherlands as the closest hit (GenBank accession: KY250316.1–250323.1) for all four samples (Fraaij et al., 2016), and were classified as FLUAV of the Eurasian avian lineage. In silico analysis of our strains predicted susceptibility to the neuraminidase inhibitors Oseltamivir, Zanamivir and Peramivir, with no pandemic classification and belonged to the global 1C.2.1 swine H1 clade. To estimate the evolutionary relationship of the obtained genomes, we carried out a phylogenetic analysis of the gene segment HA from FLUAV sequences available from swine hosts between 2010 and 2020 and from humans between 2015 and 2020. A total of 7620 sequences were initially included. A total of 127 representative non‐redundant sequences were used to generate the phylogenetic reconstruction. The evolutionary reconstruction from the generated database located our four sequences within a cluster that includes a FLUAV virus strain (KY250319) isolated from a child with a severe acute respiratory infection in the Netherlands (Fraaij et al., 2016). The phylogenetic inference depicts a clear division of the FLUAVs within two major clusters, which have human (red) and/or swine (blue) as a host (Figure 6a). Occasionally, a mixing of hosts is noted in both swine and human clades.

## 4 DISCUSSION

The European Union (EU) is the world's second biggest producer of pork after China and the biggest exporter of pork products (https://ec.europa.eu/info/food-farming-fisheries/animals-and-animal-products/animal-products/pork_en). The major production basin extends from Germany (specifically Nordrhein‐Westfalen and Niedersachsen) to Belgium (Vlaams Gewest) and accounts for 30% of EU pigs (https://ec.europa.eu/eurostat/statistics‐explained/pdfscache/3688.pdf). The large livestock population and density in areas such as these can facilitate disease transmission within herds and between livestock and humans (Kwok et al., 2020). Therefore, surveillance of livestock and the surrounding environment is a hallmark of early detection of potential epidemic/pandemic pathogens of human and animal significance.

The recent rapid technological advances and availability of NGS platforms fuel our grasp on viral diagnostics, surveillance and transmission directly from sample material. However, several wet‐lab and e‐lab hurdles remain. Sensitivity was labelled as the most pressing wet‐lab issue (Greninger, 2018). Pre‐lysis enrichment to increase sensitivity relies on microorganisms’ structural integrity (Hasan et al., 2016). However, fresh specimens are not always achievable or practical. Therefore, we compared a post‐lysis enrichment technique, ViroCap, to shotgun metagenomics (with only a simple DNase treatment) to estimate and determine its ability to detect and characterize the virome of pigs.

To determine the impact of ViroCap on sensitivity, we used paired aliquots from the same sequencing library pre‐ and post‐ViroCap. ViroCap increased the number of viral reads significantly and allowed improved detection of viruses on the read and contig level. The increased sequence depth of viral contigs improved single‐nucleotide resolution for phylogenetic and antiviral resistance analyses. However, the increased number of viral reads by ViroCap did not always result in longer viral contigs. Coverage bias of TSC methods has been reported previously (Naccache et al., 2016). The inability to yield whole genomes consistently with capture probes has also been reported previously, as probes can be less efficient in lower viral abundances due to coverage bias and bias towards viral organisms with high loads in multiplexed TSC approaches (Quick et al., 2017; Naccache et al., 2016). Overall, the use of short‐read sequencing (2 × 76 bp) could also have contributed towards shorter assemblies. The application of long‐read sequencing platforms combined with ViroCap might be an option to reduce taxonomic misassignments in the future (Schuele et al., 2020). Targeted PCR amplification has been shown to yield whole‐genomes more consistently but is dependent on primer target matches and, therefore, primarily suitable during outbreak scenarios such as Ebola (Deng et al., 2020) and SARS‐CoV‐2 (Meredith et al., 2020).

Read‐based taxonomical approaches were prone to misassignments in closely related viruses such as bat adenovirus and equine adenovirus. Viruses with high genetic diversity and recombination events, such as porcine astroviruses, also resulted in misassignments. A contig‐based approach improved taxonomical assignment but resulted in reduced sensitivity. An evaluation of different assemblers revealed that SPAdes yielded the highest number of viral contigs, whereas MEGAHIT yielded the longest contigs. Indeed, MEGAHIT was recently deemed one of the leading choices to assemble a metagenome in the Critical Assessment of Metagenome Interpretation (CAMI) challenge (Sczyrba et al., 2017).

Important respiratory swine pathogens that were detected included PRRSV, FLUAV and porcine astrovirus (PoAstV). PoAstV genotypes 2–5 have been reported in pigs with diarrhoea or respiratory symptoms and asymptomatic pigs. Interestingly, co‐infections with different genotypes have been frequently reported (Lv et al., 2019). Astroviruses show wide genetic diversity in humans and animals, indicating the possibility that astroviruses could cross the species barrier (Fischer et al., 2017). Several pig pathogens that can cause gastroenteric symptoms were also detected in NS samples within the same farm, such as swine norovirus, porcine kobuviruses, porcine sapelovirus and rotavirus. Curiously, diarrhoea was never listed as a symptom. Therefore, the relevance of these viruses within these herds is somewhat unclear. Rotavirus was detected in two samples. A previous study which investigated rotaviruses revealed potential transmission events between humans and pigs (Phan et al., 2016). However, in order to determine the zoonotic potential of this finding, more samples would need to be screened from both pigs and humans within the area. Nervous system‐related viruses that were detected included bocaviruses, mamastrovirus 2 and 3, and porcine pestivirus 1. Interestingly, these viruses were frequently co‐detected with PRRSV; however, the significance of this association remains to be ascertained.

To better understand the potential of metagenomics for clinical and public health, we studied two viruses, particularly PRRSV and FLUAV. PRRSV poses a high economic cost and remains one of the most widespread viruses in pig farms worldwide. Although it has the fastest nucleotide substitution rate of any RNA virus (up to 10^−2^ substitutions per site; Hanada et al., 2005), we could not recover such a high mutation rate. Our study's low mutation rate may be related to the fact that our samples include several vaccine‐related strains that vary little over time compared to the wild type. We also detected little variation of lineages in the studied subpopulation. Little variability and the overrepresentation of lineage 1 amongst our samples could be related to patterns of evolution and spread of vaccine‐related strains. Although we did not detect mixed clusters in the farms, it is known that the movement of piglets by trading could serve as a transmission route for PRRSV (Hanada et al., 2005).

Denmark is the lead exporter of piglets in the EU, trading mainly to Poland and the Netherlands. The latter country then trades pigs mainly to Germany for slaughtering (https://ec.europa.eu/eurostat/statistics‐explained/pdfscache/3688.pdf). These intra‐EU exchanges are reflected in the FLUAV tree, in which four closely related FLUAV strains from one German farm clustered together with strains from the Netherlands and Denmark. Interestingly, the study's closest neighbour was a strain from the Netherlands, which was reported to cause a severe acute respiratory infection in a child (Fraaij et al., 2016). At the time, the case was considered incidental and rare. However, the continuous presence of these strains in pigs should be monitored permanently as mutations (genetic drift) can occur with the potential to cause human epidemics or even pandemics (Nava et al., 2009). Zoonotic infections with influenza A swine H1_av_N1 have been reported in Germany in 2020 (Dürrwald et al., 2020). Furthermore, another plausible scenario is a possible establishment of FLUAV reassorting viruses (e.g. Clade 1A.3.3.2) that have enhanced transmission to humans, which has occurred in several provinces in China since 2014 (Sun et al., 2020). Thus, generating (nearly) complete viral genomes directly from sample material could reveal strains that may have acquired antigenic changes increasing their zoonotic potential (Dürrwald et al., 2020). Although the infectivity potential of a particular viral strain does not determine the susceptibility of the host, the complete genome of viruses can help with the in silico prediction of enhanced human receptor binding and specificity, which can be tested experimentally in cells expressing human receptors (Schmier et al., 2015).

Limitations of this study include the pre‐selection of farms based on their ability to enable the long‐term monitoring of FLUAV, PRRSV and *Salmonella* within the Food Protects project. Therefore, direct epidemiological links between farms were not feasible. Additionally, although DNase treatment increases the viral sensitivity, DNA viruses which are not expressing at the time of sampling could not be detectable. Finally, although the pooling of samples permits an efficient screening of circulating viruses on a herd level, detections are unable to be linked to an individual animal.

In conclusion, sequencing of both SISPA‐derived and viral‐enriched cDNA has revealed a rather intricate co‐infection pattern within sick and healthy pigs. Identifying viruses directly from sample material allows hypothesis‐free detection and characterization of unexpected pathogens. TSC increased viral sensitivity and genome coverage for most viruses, facilitating future applications of viral *quasispecies* detection and antiviral therapy. The increased viral sensitivity of ViroCap did not always result in whole‐genome sequences. Within our sample cohort, SPAdes was the best choice for detecting viruses, whereas MEGAHIT yielded the longest contigs. Understanding the swine virome and the potential zoonotic pathogens present within these crucial mixing vessels will allow for better outbreak preparedness in livestock disease and subsequent human transmission.

## CONFLICT OF INTERESTS

John W. A. Rossen is employed by IDbyDNA. Silke Peter consults for IDbyDNA. This did not influence the interpretation of reviewed data and conclusions drawn nor the drafting of the manuscript, and no support was obtained from them. All other authors declare no conflict of interest.

## ETHICS

The sampling within the Food Protects project has been classified as an animal study and was approved on the 22.09.2017 by the respective state office for nature, environment and consumer protection (file reference: 84.02.05.40.17.079).

## Supporting information

Supporting Information

## Data Availability

All sequencing data have been deposited in at the Sequence Read Archive under the BioProject number: PRJNA701157.
