# Supplementary material for: Application of shotgun metagenomics sequencing and targeted sequence capture to detect circulating porcine viruses in the Dutch–German border region
Source: Transbound Emerg Dis. 2021 Aug 28;69(4):2306–19. doi: 10.1111/tbed.14249 (PMC9540031; doi:10.1111/tbed.14249)
Supplement: Supplementary file 1 — Supporting Information [file TBED-69-2306-s001.docx]

**SUPPORTING INFORMATION**

Table S1. Overview of all detected viruses within the herds and farms using the read-based tool Taxonomer.

| **Farm number-herd & Sample ID** | **Reference name** | **Symptoms & sample type** | **Age** | **Herd size** |
| --- | --- | --- | --- | --- |
| **1-1 (251-1)** | Atypical porcine pestivirus 1 | No symptoms BS | Pre-fattening | 3300 |
|  | Porcine kobuvirus |  |  |  |
|  | Porcine kobuvirus SH-W-CHN/2010/China |  |  |  |
|  | Porcine pestivirus 1 |  |  |  |
| **1-2  (251-2)** | Atypical porcine pestivirus 1 | No symptoms BS | Pre-fattening | 3300 |
| **1-3 (251-3)** | PRRSV | No symptoms BS | Pre-fattening | 3300 |
|  | Mamastrovirus 2 |  |  |  |
| **2-1 (254-3)** | PRRSV | No symptoms BS | Pre-fattening | 1900 |
| **2-2 (254-5)** | PRRSV | No symptoms BS | Pre-fattening | 1900 |
| **3-1 (278-1)** | PRRSV | Respiratory BS | Pre-fattening | 230 |
|  | Porcine astrovirus 2 |  |  |  |
| **3-2 (278-10)** | Atypical porcine pestivirus 1 | Respiratory BS | Pre-fattening | 230 |
|  | PRRSV (Lelystad) virus |  |  |  |
| **3-3 (278-3)** | Astrovirus wild boar/WBAstV-1/2011/HUN | Respiratory BS | Pre-fattening | 230 |
|  | Hubei tombus-like virus 8 |  |  |  |
|  | PRRSV |  |  |  |
| **3-4 (278-4)** | Astrovirus wild boar/WBAstV-1/2011/HUN | Respiratory BS | Pre-fattening | 230 |
|  | PRRSV |  |  |  |
| **3-5 (278-5)** | Astrovirus wild boar/WBAstV-1/2011/HUN | Respiratory BS | Pre-fattening | 230 |
|  | Atypical porcine pestivirus 1 |  |  |  |
|  | PRRSV |  |  |  |
|  | Pasivirus A1 |  |  |  |
| **3-6 (278-6)** | Astrovirus wild boar/WBAstV-1/2011/HUN | Respiratory BS | Pre-fattening | 230 |
| **3-7 (278-7)** | Porcine astrovirus 4 | Respiratory BS | Pre-fattening | 230 |
|  | Torque teno sus virus k2a |  |  |  |
| **3-8 (278-8)** | PRRSV | Respiratory BS | Pre-fattening | 230 |
| **3-9 (278-9)** | Astrovirus wild boar/WBAstV-1/2011/HUN | Respiratory BS | Pre-fattening | 230 |
|  | Porcine astrovirus 4 |  |  |  |
|  | PRRSV |  |  |  |
| **4-1 (313-10)** | Porcine hokovirus | NA BS | Pre-fattening | NA |
|  | Ungulate tetraparvovirus 3 |  |  |  |
|  | PRRSV |  |  |  |
| **4-2 (313-11)** | Porcine hokovirus | NA BS | Mid-fattening | NA |
|  | Ungulate tetraparvovirus 3 |  |  |  |
|  | PRRSV |  |  |  |
| **4-3 (313-12)** | Parvovirus YX-2010/CHN | NA BS | Mid-fattening | NA |
| **4-4 (313-13)** | Torque teno sus virus 1b | NA BS | Mid-fattening | NA |
|  | PRRSV |  |  |  |
| **4-5 (313-6)** | PRRSV | NA BS | Pre-fattening | NA |
|  | Porcine bocavirus H18 |  |  |  |
|  | Porcine hokovirus |  |  |  |
|  | Torque teno sus virus 1b |  |  |  |
|  | Torque teno sus virus k2a |  |  |  |
| **4-6 (313-7)** | PRRSV | NA BS | Pre-fattening | NA |
|  | Parvovirus YX-2010/CHN |  |  |  |
|  | Porcine hokovirus |  |  |  |
| **4-7 (313-9)** | PRRSV | NA BS | Pre-fattening | NA |
| **5-1  (213-14)** | Astrovirus wild boar/WBAstV-1/2011/HUN | Respiratory- enteric (closed system)  -  NS | Pre-fattening | NA |
|  | Enterovirus G |  |  |  |
|  | Influenza A virus (H1N1) |  |  |  |
|  | Porcine sapelovirus 1 |  |  |  |
|  | Posavirus 1 |  |  |  |
|  | PRRSV |  |  |  |
|  | Porcine astrovirus 2 |  |  |  |
|  | Porcine astrovirus 4 |  |  |  |
|  | Porcine astrovirus 5 |  |  |  |
|  | Porcine bocavirus 5/JS677 |  |  |  |
|  | Porcine enterovirus 9 |  |  |  |
|  | Porcine kobuvirus SH-W-CHN/2010/China |  |  |  |
|  | Rotavirus A |  |  |  |
|  | Teschovirus A |  |  |  |
| **5-2  (213-15)** | Astrovirus wild boar/WBAstV-1/2011/HUN | Respiratory- enteric (closed system)  -  NS | Pre-fattening | NA |
|  | Influenza A virus (H1N1) |  |  |  |
|  | Mamastrovirus 2 |  |  |  |
|  | Pasivirus A1 |  |  |  |
|  | Porcine astrovirus 4 |  |  |  |
|  | Porcine kobuvirus |  |  |  |
|  | PRRSV |  |  |  |
| **5-3  (213-16)** | Astrovirus wild boar/WBAstV-1/2011/HUN | Respiratory- enteric (closed system)  -  NS | Mid-fattening | NA |
|  | Influenza A virus (H1N1) |  |  |  |
|  | Porcine astrovirus 4 |  |  |  |
|  | Porcine kobuvirus |  |  |  |
|  | Porcine sapelovirus 1 |  |  |  |
|  | Porcine torovirus |  |  |  |
|  | Teschovirus A |  |  |  |
|  | PRRSV |  |  |  |
| **5-4  (213-17)** | Astrovirus wild boar/WBAstV-1/2011/HUN | Respiratory- enteric (closed system)  -  NS | Mid-fattening | NA |
|  | Influenza A virus (H1N1) |  |  |  |
|  | Mamastrovirus 2 |  |  |  |
|  | Mamastrovirus 3 |  |  |  |
|  | Norovirus pig/GII/Ch6/China/2009 |  |  |  |
|  | Porcine astrovirus 2 |  |  |  |
|  | Porcine astrovirus 4 |  |  |  |
|  | Porcine kobuvirus |  |  |  |
|  | Porcine sapelovirus 1 |  |  |  |
|  | Teschovirus A |  |  |  |
| **6-1 (213-19)** | Atypical porcine pestivirus 1 | Respiratory BS | Piglets (20 kg) | NA |
|  | PRRSV |  |  |  |
| **6-2 (213-20)** | Atypical porcine pestivirus 1 | Respiratory BS | Piglets (20 kg) | NA |
|  | PRRSV |  |  |  |
|  | Parvovirus YX-2010/CHN |  |  |  |
|  | Rotavirus C |  |  |  |
| **7-1 (213-21)** | Atypical porcine pestivirus 1 | NA BS | Piglets (9-11 & 13 weeks) | NA |
|  | Porcine astrovirus 4 |  |  |  |
|  | PRRSV |  |  |  |
| **7-2 (213-22)** | Atypical porcine pestivirus 1 | NA BS | Piglets (9-11 & 13 weeks) | NA |
|  | Porcine pestivirus 1 |  |  |  |
|  | PRRSV |  |  |  |
| **8-1 (213-23)** | PRRSV | NA BS | Piglets (9-11 & 13 weeks) | NA |
| **8-2 (213-24)** | PRRSV | NA BS | Piglets (15 - 20 kg) | NA |
|  | Porcine bocavirus H18 |  |  |  |
|  | Porcine respirovirus 1 |  |  |  |
|  | Rotavirus C |  |  |  |
| **8-3 (213-25)** | Mamastrovirus 2 | NA BS | Piglets (15 - 20 kg) | NA |
|  | Rotavirus C |  |  |  |
|  | PRRSV |  |  |  |
| **9-1 (213-26)** | Porcine torovirus | Respiratory BS | Pre-fattening | NA |
|  | Ungulate tetraparvovirus 3 |  |  |  |
| **9-2 (213-27)** | PRRSV | Respiratory BS | Pre-fattening | NA |
| **1-1 (251-1)** | Atypical porcine pestivirus 1 | No symptoms BS | Pre-fattening | 3300 |
|  | Porcine kobuvirus |  |  |  |
|  | Porcine kobuvirus SH-W-CHN/2010/China |  |  |  |
|  | Porcine pestivirus 1 |  |  |  |
| **1-2  (251-2)** | Atypical porcine pestivirus 1 | No symptoms BS | Pre-fattening | 3300 |
| **1-3 (251-3)** | PRRSV | No symptoms BS | Pre-fattening | 3300 |
|  | Mamastrovirus 2 |  |  |  |
| **2-1 (254-3)** | PRRSV | No symptoms BS | Pre-fattening | 1900 |
| **2-2 (254-5)** | PRRSV | No symptoms BS | Pre-fattening | 1900 |
| **3-1 (278-1)** | PRRSV | Respiratory BS | Pre-fattening | 230 |
|  | Porcine astrovirus 2 |  |  |  |
| **3-2 (278-10)** | Atypical porcine pestivirus 1 | Respiratory BS | Pre-fattening | 230 |
|  | PRRSV (Lelystad) virus |  |  |  |
| **3-3 (278-3)** | Astrovirus wild boar/WBAstV-1/2011/HUN | Respiratory BS | Pre-fattening | 230 |
|  | Hubei tombus-like virus 8 |  |  |  |
|  | PRRSV |  |  |  |
| **3-4 (278-4)** | Astrovirus wild boar/WBAstV-1/2011/HUN | Respiratory BS | Pre-fattening | 230 |
|  | PRRSV |  |  |  |
| **3-5 (278-5)** | Astrovirus wild boar/WBAstV-1/2011/HUN | Respiratory BS | Pre-fattening | 230 |
|  | Atypical porcine pestivirus 1 |  |  |  |
|  | PRRSV |  |  |  |
|  | Pasivirus A1 |  |  |  |
| **3-6 (278-6)** | Astrovirus wild boar/WBAstV-1/2011/HUN | Respiratory BS | Pre-fattening | 230 |
| **3-7 (278-7)** | Porcine astrovirus 4 | Respiratory BS | Pre-fattening | 230 |
|  | Torque teno sus virus k2a |  |  |  |
| **3-8 (278-8)** | PRRSV | Respiratory BS | Pre-fattening | 230 |
| **3-9 (278-9)** | Astrovirus wild boar/WBAstV-1/2011/HUN | Respiratory BS | Pre-fattening | 230 |
|  | Porcine astrovirus 4 |  |  |  |
|  | PRRSV |  |  |  |
| **4-1 (313-10)** | Porcine hokovirus | NA BS | Pre-fattening | NA |
|  | Ungulate tetraparvovirus 3 |  |  |  |
|  | PRRSV |  |  |  |
| **4-2 (313-11)** | Porcine hokovirus | NA BS | Mid-fattening | NA |
|  | Ungulate tetraparvovirus 3 |  |  |  |
|  | PRRSV |  |  |  |
| **4-3 (313-12)** | Parvovirus YX-2010/CHN | NA BS | Mid-fattening | NA |
| **4-4 (313-13)** | Torque teno sus virus 1b | NA BS | Mid-fattening | NA |
|  | PRRSV |  |  |  |
| **4-5 (313-6)** | PRRSV | NA BS | Pre-fattening | NA |
|  | Porcine bocavirus H18 |  |  |  |
|  | Porcine hokovirus |  |  |  |
|  | Torque teno sus virus 1b |  |  |  |
|  | Torque teno sus virus k2a |  |  |  |
| **4-6 (313-7)** | PRRSV | NA BS | Pre-fattening | NA |
|  | Parvovirus YX-2010/CHN |  |  |  |
|  | Porcine hokovirus |  |  |  |
| **4-7 (313-9)** | PRRSV | NA BS | Pre-fattening | NA |
|  | Parvovirus YX-2010/CHN |  |  |  |
|  | Porcine bocavirus H18 |  |  |  |
|  | Porcine hokovirus |  |  |  |

Abbreviations: BS, blood serum; NA, not available; NS, nasal swab; PRRSV, porcine reproductive and respiratory syndrome virus.

**Table S2.** Total sequence reads and proportion of viral reads in SISPA and ViroCap derived sequencing libraries. Data was obtained using Taxonomer. The average number of sequencing reads for SISPA derived sequencing libraries was 27.5 Gbp and 33 Gbp for ViroCap derived sequencing libraries.

| **Sample ID** | **SISPA** | | **ViroCap** | |  | |
| --- | --- | --- | --- | --- | --- | --- |
|  | **Total reads (Gbp)** | **Viral reads (%)** | **Total reads (Gbp)** | **Viral reads (%)** | **Fold change (viral)** |  |
| 213-14 | 30.1 | 2.41 | 60.2 | 57.86 | 24.0 |  |
| 213-15 | 27.9 | 0.79 | 30.6 | 32.57 | 41.0 |  |
| 213-16 | 28.0 | 0.01 | 21.3 | 0.51 | 38.7 |  |
| 213-17 | 31.5 | 0.07 | 22.4 | 4.31 | 57.9 |  |
| 213-19 | 23.1 | 6.35 | 35.4 | 37.47 | 5.9 |  |
| 213-20 | 14.7 | 0.80 | 11.1 | 48.67 | 60.5 |  |
| 213-21 | 28.4 | 0.14 | 20.7 | 1.42 | 10.4 |  |
| 213-22 | 23.0 | 3.69 | 23.3 | 19.19 | 5.2 |  |
| 213-23 | 26.1 | 5.59 | 38.5 | 30.76 | 5.5 |  |
| 213-24 | 21.2 | 1.41 | 21.1 | 63.45 | 45.0 |  |
| 213-25 | 28.5 | 0.36 | 20.7 | 2.67 | 7.4 |  |
| 213-26 | 21.3 | 1.11 | 1.0 | 20.60 | 18.6 |  |
| 213-27 | 29.1 | 1.84 | 24.3 | 8.45 | 4.6 |  |
| 251-1 | 19.7 | 0.47 | 10.7 | 1.93 | 4.1 |  |
| 251-2 | 40.1 | 0.05 | 9.4 | 0.32 | 6.5 |  |
| 251-3 | 49.8 | 0.34 | 30.1 | 55.81 | 162.8 |  |
| 251-4 | 8.2 | 0.41 | 3.7 | 1.54 | 3.8 |  |
| 251-5 | 28.4 | 0.32 | 9.7 | 24.75 | 76.4 |  |
| 254-3 | 39.9 | 1.75 | 37.1 | 63.01 | 36.0 |  |
| 254-5 | 22.6 | 1.83 | 24.6 | 66.19 | 36.3 |  |
| 278-1 | 6.4 | 0.39 | 5.9 | 31.06 | 79.8 |  |
| 278-10 | 20.1 | 1.22 | 14.2 | 24.40 | 20.1 |  |
| 278-3 | 35.4 | 3.20 | 40.2 | 95.66 | 29.9 |  |
| 278-4 | 29.5 | 4.98 | 19.5 | 76.69 | 15.4 |  |
| 278-5 | 24.8 | 0.29 | 9.0 | 20.46 | 71.6 |  |
| 278-6 | 24.8 | 1.36 | 14.9 | 57.83 | 42.4 |  |
| 278-7 | 23.8 | 1.89 | 42.5 | 61.88 | 32.8 |  |
| 278-8 | 24.6 | 1.20 | 8.8 | 10.07 | 8.4 |  |
| 278-9 | 26.9 | 4.70 | 123.5 | 84.01 | 17.9 |  |
| 313-10 | 51.4 | 1.29 | 51.2 | 80.21 | 62.3 |  |
| 313-11 | 42.3 | 1.16 | 21.8 | 62.94 | 54.1 |  |
| 313-12 | 21.6 | 1.29 | 36.4 | 75.94 | 59.1 |  |
| 313-13 | 37.2 | 1.12 | 8.2 | 32.87 | 29.4 |  |
| 313-6 | 26.6 | 1.36 | 15.8 | 59.00 | 43.4 |  |
| 313-7 | 22.2 | 1.07 | 16.3 | 57.46 | 53.6 |  |
| 313-9 | 29.9 | 1.19 | 8.9 | 11.37 | 9.5 |  |

Abbreviations: Gbp, Giga base pairs.

**Table S3.** Relationship between FLUAV Ct values with the proportion of viral reads in SISPA and ViroCap based sequencing libraries of nasal swab pooled samples (n=4).

| **Sample** | **Ct-value** | **SISPA** | | | **ViroCap** | | |
| --- | --- | --- | --- | --- | --- | --- | --- |
|  |  | **Total reads** | **FLUAV reads** | **FLUAV**  **(%)** | **Total reads** | **FLUAV reads** | **FLUAV(%)** |
| 213-14 | FLUAV **(Ct-19)** | 30,137,906 | 582,812 | **1.934%** | 60,152,651 | 30,376,429 | **50.499%** |
| 213-15 | FLUAV **(Ct-20)** | 27,916,018 | 182,694 | **0.654%** | 30,615,400 | 8,696,337 | **28.405%** |
| 213-16 | FLUAV **(Ct-22)** | 31,480,584 | 17,154 | **0.054%** | 22,449,234 | 847,690 | **3.776%** |
| 213-17 | FLUAV **(Ct-26)** | 28,036,475 | 1,340 | **0.005%** | 21,347,420 | 94,154 | **0.441%** |

Abbreviations: FLUAV, influenza A virus.

**Table S4.** Best hits of rotavirus A segments detected in sample 213-24.

| **Gene** | **Consensus (mapping)** | **Genome coverage** | [**Description**](https://blast.ncbi.nlm.nih.gov/Blast.cgi) | [**Ident**](https://blast.ncbi.nlm.nih.gov/Blast.cgi?CMD=Get&ADV_VIEW=yes&ADV_VIEW=on&ALIGNMENTS=100&ALIGNMENT_VIEW=Pairwise&CONFIG_DESCR=2,3,6,7,8,9,10,11,12&DATABASE_SORT=0&DESCRIPTIONS=100&DYNAMIC_FORMAT=on&FIRST_QUERY_NUM=0&FORMAT_NUM_ORG=1&FORMAT_OBJECT=Alignment&FORMAT_PAGE_TARGET=&FORMAT_TYPE=HTML&GET_SEQUENCE=yes&I_THRESH=&LINE_LENGTH=60&MASK_CHAR=2&MASK_COLOR=1&NUM_OVERVIEW=100&PAGE=MegaBlast&QUERY_INDEX=0&QUERY_NUMBER=0&RESULTS_PAGE_TARGET=&RID=B1W4CKZH013&SHOW_LINKOUT=yes&SHOW_OVERVIEW=yes&STEP_NUMBER=&ADV_VIEW=on&DISPLAY_SORT=3&HSP_SORT=3)**ity** | **GenBank accession number** | **Description** |
| --- | --- | --- | --- | --- | --- | --- |
| VP1 | 615bp | 19.2% | Rotavirus A gene for structural protein VP1, strain: RVA/Human-wt/PHL/TGE13-39/2013/G4P[6] | 93.67% | LC061616 | Genetic diversity of group A rotaviruses detected from  environmental and clinical samples, including a porcine-like human rotaviruses detected from a child in the Philippines |
| VP2 | 1168bp | 43.7% | Rotavirus A strain RVA/Human-wt/HUN/BP1125/2004/G4P6/VP2 VP2 gene | 91.96% | KF835898 | Zoonotic transmission of reassortant porcine G4P[6] rotaviruses in Hungarian pediatric patients identified sporadically over a 15 year period |
| VP3 | 1348bp | 52% | Rotavirus A Hu/BEL/BE2001/2009/G9P[6] VP3 gene | 93.62% | JQ993323 | Genetic diversity of group A rotaviruses detected from environmental and clinical samples, including a porcine-like human rotaviruses detected from a child in the Philippines |
| VP4 | - |  | - | - | - |  |
| VP6 | - |  | - | - | - |  |
| VP7 | 156bp | 14.7% | KC254780.1 Porcine rotavirus A strain PGRV11 structural protein VP7 (VP7) gene | 95.04% | JX498942 | Molecular epidemiology of porcine rotavirus strains with diarrhea in Mainland China |

**Table S5**. Viruses found in nasal swabs sample pools (n=4) of pigs suffering from respiratory syndromes by aligning the contigs to the NCBI database with BLASTn.

| **Virus** | **Proportion of positive sample pools (%)** |
| --- | --- |
| Astrovirus wild boar/WBAstV-1/2011/HUN | 100% |
| Enterovirus G | 25% |
| Influenza A virus (FLUAV) | 100% |
| PRRSV | 75% |
| Mamastrovirus 2 | 50% |
| Mamastrovirus 3 | 25% |
| Norovirus GII.2 | 25% |
| Pasivirus A1 | 25% |
| Porcine astrovirus 2 | 25% |
| Porcine astrovirus 4 | 75% |
| Porcine astrovirus 5 | 25% |
| Porcine enterovirus 9 | 25% |
| Porcine kobuvirus | 100% |
| Porcine sapelovirus 1 | 100% |
| Porcine torovirus | 25% |
| Posavirus 1 | 25% |
| Rotavirus A | 25% |
| Teschovirus A | 75% |

Abbreviations: PRRSV, porcine reproductive and respiratory syndrome virus.

**Table S6.** Viruses found in blood serum sample pools (n=13) of pigs suffering from respiratory syndromes by aligning the contigs to the NCBI database with BLASTn.

| **Virus** | **Proportion of positive sample pools (%)** |
| --- | --- |
| Astrovirus wild boar/WBAstV-1/2011/HUN | 38% |
| Atypical porcine pestivirus 1 | 15% |
| Hubei tombus-like virus 8 | 8% |
| PRRSV | 62% |
| Pasivirus A1 | 8% |
| Porcine astrovirus 2 | 8% |
| Porcine astrovirus 4 | 15% |
| Porcine torovirus | 8% |
| Torque teno sus virus k2a | 8% |
| Ungulate tetraparvovirus 3 | 8% |

Abbreviations: PRRSV, porcine reproductive and respiratory syndrome virus.

**Table S7**. Viruses found in blood serum sample pools (n=5) obtained from pigs without symptoms by aligning the contigs to the NCBI database with BLASTn.

| **Virus** | **Proportion of positive sample pools (%)** |
| --- | --- |
| Porcine pestivirus 1 | 40% |
| Porcine kobuvirus | 40% |
| PRRSV | 60% |
| Mamastrovirus 2 | 20% |

Abbreviations: PRRSV, porcine reproductive and respiratory syndrome virus.

**Table S8.** Additional complete or near-complete viral contigs obtained in this study and identity to the closest hit (NCBI GenBank).

| **Sample** | **Best hit (NCBI BLASTn)** | [**Ident**](https://blast.ncbi.nlm.nih.gov/Blast.cgi?CMD=Get&ADV_VIEW=yes&ADV_VIEW=on&ALIGNDB_BATCH_ID=589740482&ALIGNDB_CGI_HOST=blast.be-md.ncbi.nlm.nih.gov&ALIGNDB_CGI_PATH=/ALIGNDB/alndb_asn.cgi&ALIGNDB_MASTER_ALIAS=SD_ALIGNDB_MASTER&ALIGNDB_MAX_ROWS=100&ALIGNDB_ORDER_CLAUSE=seq_evalue%20asc,aln_id%20asc&ALIGNDB_WHERE_CLAUSE=seq_evalue%20is%20not%20null&ALIGNMENTS=100&ALIGNMENT_VIEW=Pairwise&CONFIG_DESCR=2,3,6,7,8,9,10,11,12&DATABASE_SORT=0&DESCRIPTIONS=100&DYNAMIC_FORMAT=on&FIRST_QUERY_NUM=0&FORMAT_NUM_ORG=1&FORMAT_OBJECT=Alignment&FORMAT_PAGE_TARGET=&FORMAT_TYPE=HTML&GET_SEQUENCE=yes&I_THRESH=&LINE_LENGTH=60&MASK_CHAR=2&MASK_COLOR=1&NUM_OVERVIEW=100&PAGE=MegaBlast&QUERY_INDEX=0&QUERY_NUMBER=0&RESULTS_PAGE_TARGET=&RID=AZ491Z9F013&SHOW_LINKOUT=yes&SHOW_OVERVIEW=yes&STEP_NUMBER=&USE_ALIGNDB=true&ADV_VIEW=on&DISPLAY_SORT=3&HSP_SORT=3)**ity (best hit)** | **GenBank accession number of the best hit** | **Genome coverage (best hit)** | **Contig length** | **GenBank accession number** |
| --- | --- | --- | --- | --- | --- | --- |
| 213-14 | Porcine kobuvirus SH-W-CHN/2010/China | 89.84% | LC210616 | 100% | 8245 | MZ334483 |
| 213-20 | Atypical pestivirus | 94.20% | KU041639 | 100% | 10908 | MZ334484 |
| 213-24 | Porcine respirovirus 1  (porcine parainfluenza virus 1) | 96.05% | JX857410 | 99.7% | 15344 | MT995732 |
| 278-5 | Pasivirus A1 | 83.35% | LT898422 | 99.3% | 6855 | Submitted |
| 278-5 | Atypical pestivirus | 93.46% | MH885413 | 94.6% | 10877 | Submitted |
| 313-11 | Porcine hokovirus | 99.52% | JF738366 | 100% | 5095 | Submitted |
| 313-12 | Ungulate tetraparvovirus 3 | 98.05% | KX517759 | 95.4% | 5281 | Submitted |


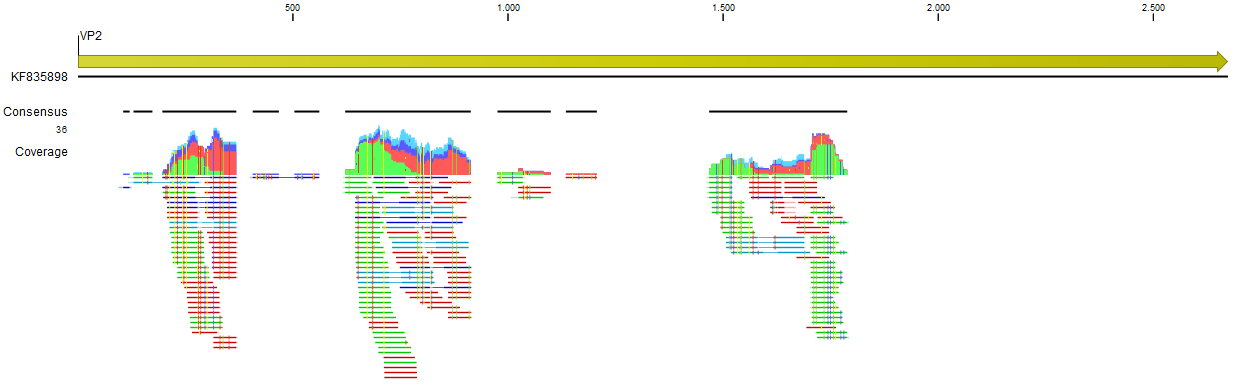


**Figure S1**. VP2 segment coverage and read depth obtained by mapping the reads of sample 213-24 against the NCBI best hit (GenBank accession number KF835898) rotavirus using CLC Genomics Workbench.


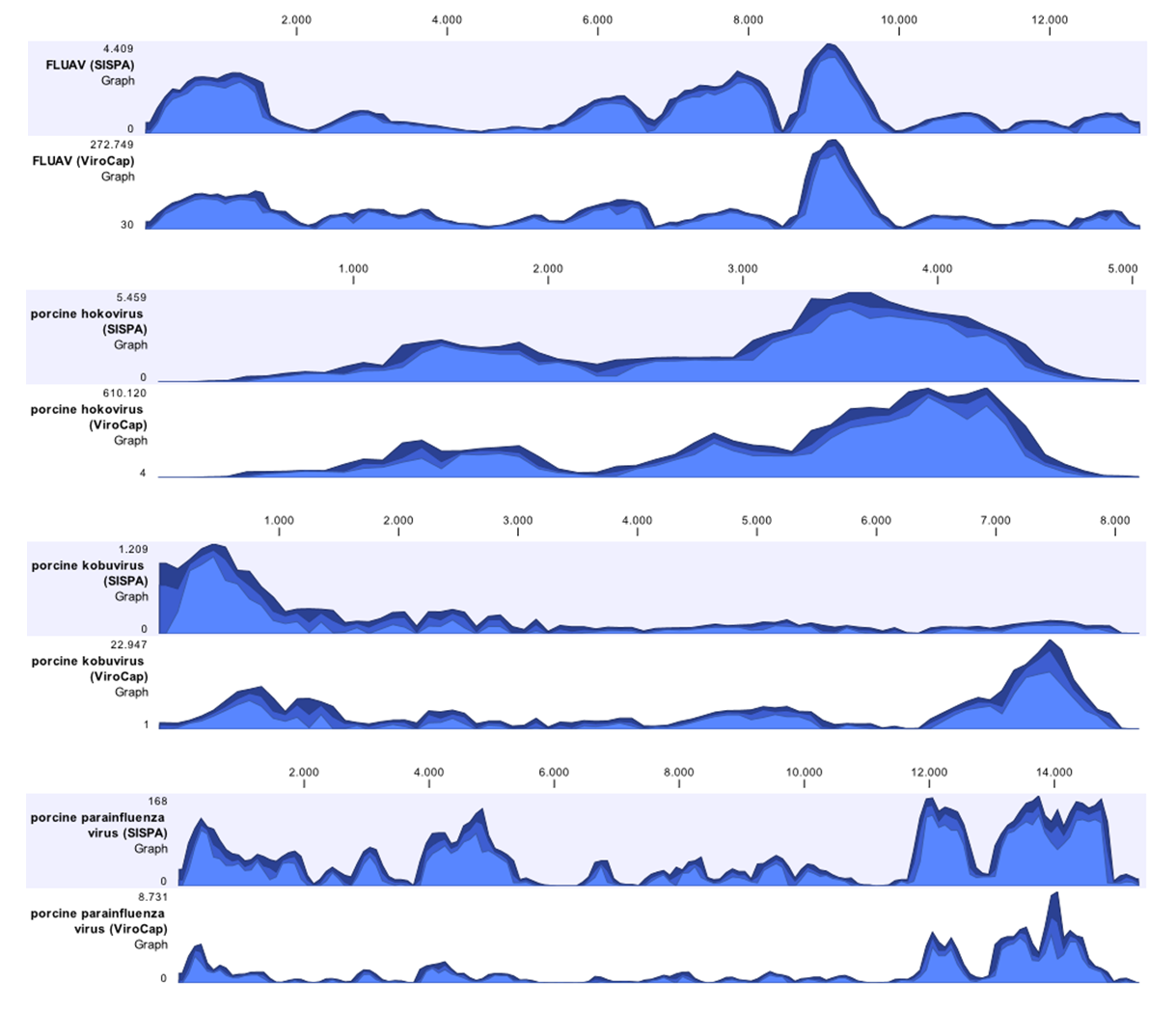


**Figure S2**. Examples of a genome-wide comparison of sequence coverage of several viral genomes using SISPA and ViroCap. The sequencing depth coverage is shown in blue.
